# Anti-metastatic Properties of Naproxen-HBTA in a Murine Model of Cutaneous Melanoma

**DOI:** 10.3389/fphar.2019.00066

**Published:** 2019-02-08

**Authors:** Giuseppe Ercolano, Paola De Cicco, Francesco Frecentese, Irene Saccone, Angela Corvino, Flavia Giordano, Elisa Magli, Ferdinando Fiorino, Beatrice Severino, Vincenzo Calderone, Valentina Citi, Giuseppe Cirino, Angela Ianaro

**Affiliations:** ^1^Department of Pharmacy, University of Naples Federico II, Naples, Italy; ^2^Department of Pharmacy, University of Pisa, Pisa, Italy

**Keywords:** cyclooxygenases (COXs), hydrogen sulfide (H_2_S), non-steroidal anti-inflammatory drugs (NSAIDs), melanoma, target-therapy, apoptosis

## Abstract

The beneficial effects of H_2_S-release and of COXs-inhibition have been exploited in the design of novel anti-inflammatory drugs, the H_2_S-releasing non-steroidal anti-inflammatory drugs (H_2_S-NSAIDs), showing promising potential for chemoprevention in cancers. Here, we evaluated the efficacy of a new H_2_S-releasing derivative of naproxen, named naproxen-4-hydroxybenzodithioate (naproxen-HBTA), in reducing metastatic melanoma features, both *in vitro* and *in vivo*. The novel H_2_S donor has been prepared following a synthetic scheme that provided high yields and purity. In particular, we investigated the effect of naproxen-HBTA *in vitro* on several metastatic features of human melanoma cells such as proliferation, migration, invasion, and colonies formation and *in vivo* in a model of cutaneous melanoma. Cell culture studies demonstrated that naproxen-HBTA induced caspase 3-mediated apoptosis and inhibited motility, invasiveness, and focus formation. Finally, daily oral treatment with naproxen-HBTA significantly suppressed melanoma growth and progression in mice. In conclusion, by using this dual approach we propose that the COX-2 and H_2_S pathways could be regarded as novel therapeutic targets/tools to generate new treatment options based on “combination therapy” for melanoma.

## Introduction

Standard treatment of metastatic melanoma allows tumor remission to be achieved only in a small subset of patients. A recent network meta-analysis including 28,561 patients with unresectable lymph node metastasis and distant metastatic cutaneous melanoma reported that, among the several systemic treatments, the most effective strategies in terms of efficacy and progression-free survival were represented by immune checkpoint inhibitors (anti-PD-1 monoclonal antibodies) and small molecule targeted drugs. These data confirm that the targeted approach in malignant melanoma treatment is successful (Pasquali et al., 2018). Actually, the most clinically used small molecule targeted drugs are the specific BRAF, MEK, or c-KIT inhibitors but, unfortunately, resistance develops invariably (Sun et al., 2014).

Studies on melanoma molecular pathogenesis led to the identification of new pathways and molecular targets. Recently, we and others have proposed a key role for cyclooxygenase (COX)-2 pathway in melanoma development and progression (Kuzbicki et al., 2006; Chwirot and Kuzbicki, 2007; Becker et al., 2009; Panza et al., 2016, 2018). A direct relationship between COX-2 expression and cancer incidence has already been shown in various tumor types, as well as increased tumorigenesis after genetic manipulation of COX-2. Moreover, significant anti-tumor properties of non-steroidal anti-inflammatory drugs (NSAIDs) have been demonstrated in animal models and in some human cancers (Ruegg et al., 2003; Trifan and Hla, 2003; Sobolewski et al., 2010). Furthermore, NSAIDs have also shown therapeutic activity in UV-induced skin tumors in mice (Fischer et al., 2003).

In this paper, by using *in vitro* and *in vivo* approaches, we evaluated the efficacy of a new COXs inhibitor naproxen-4-hydroxybenzodithioate (naproxen-HBTA) in reducing melanoma development and progression. Naproxen-HBTA has been synthesized by esterification of commercially available naproxen with HBTA, a compound identified by our research group as a new efficient hydrogen sulfide (H_2_S) donor described for this effect for the first time here. The novel H_2_S donor has been prepared following an innovative procedure that represents an easier route to access to aromatic dithioate hybrid drugs opening to the possibility of coupling the biological effects of this new hydrogen sulfide donor to already marketed drugs.

Hydrogen sulfide is an endogenous gasotransmitter with a plethora of cellular and molecular targets that has been recently demonstrated to be involved in human melanoma progression (Panza et al., 2015).

Our study demonstrates that naproxen-HBTA is more effective in inhibiting melanoma proliferation, migration, invasion, and colony formation *in vitro* as well as tumor development *in vivo* then the parent drug naproxen. Thus, by using this dual approach we propose that COX-2 and H_2_S pathway could be innovative therapeutic targets/tools to generate new treatment options based on “combination therapy.”

## Materials and Methods

### Reagents

All reagents, solvents or other chemicals were commercial products purchased from Sigma-Aldrich. All reactions were followed by TLC carried out on Merk silica gel 60 F254 plates with fluorescent indicator on the plates were visualized with UV light (254 nm). Preparative chromatographic purifications were performed using silica gel column (Kieselgel 60). Microwave reactions were performed using a microwave oven (ETHOS 1600, Milestone) especially designed for organic synthesis. Solutions were concentrated with a Buchi R-114 rotary evaporator at low pressure. Elemental analyses were carried out on Carlo Erba model 1106; analyses indicated by the symbols of the elements were within ± 0.4% of the theoretical values. Melting points, determined using a Buchi Melting Point B-540 instrument, are uncorrected and represent values obtained on re-crystallized or chromatographically purified material. Mass spectra of intermediates and of the final product were performed on API 2000 Applied Biosystem mass spectrometer. ^1^H-NMR and ^13^C-NMR spectra were recorded on Varian Mercury Plus 400 MHz instrument. Chemical shift are reported in ppm. The following abbreviations are used to describe peak patterns when appropriate: s (singlet), d (doublet), t (triplet), m (multiplet), bs (broad singlet).

### H_2_S Determination

The characterization of the H_2_S-generating properties of HBTA has been carried out by amperometric approach, through an Apollo-4000 Free Radical Analyzer (WPI) detector and H_2_S-selective minielectrodes (ISO-H2S-2, WPI) endowed with gas-permeable membranes. The experiments were carried out at room temperature. Following the manifacturer’s instructions, a “PBS buffer 10x” was prepared (NaH_2_PO_4_.H_2_O 1.28 g, Na_2_HPO_4_.12H_2_O 5.97 g, NaCl 43.88 g in 500 mL H_2_O) and stocked at 4°C. Immediately before the experiments, the “PBS buffer 10x” was diluted in distilled water (1:10), to obtain the assay buffer (AB); pH was adjusted to 7.4. The H_2_S-selective minielectrode was equilibrated in 2 mL of the AB, until the recovery of a stable baseline. Then, 20 μL of a dimethyl sulfoxide (DMSO) solution of the H_2_S-releasing compound (HBTA) was added (final concentration of HBTA 1 mM; final concentration of DMSO in the AB 1%). The generation of H_2_S was observed for 30 min. When required by the experimental protocol, L-Cysteine 4 mM was added, before the H_2_S-donor. The correct relationship between the amperometric current (recorded in pA) and the corresponding concentration of H_2_S was determined by opportune calibration curves, which were previously obtained by the use of increasing concentrations of NaHS (1, 3, 5, 10 μM) at pH 4.0. The curves relative to the progressive increase of H_2_S vs. time, following the incubation of the tested compound, were analyzed by computer fitting procedure (software: GraphPad Prism 6.0). The parameter of Cmax (the highest concentration recorded during the recording time) and TCM50 (time required to reach a concentration = ½ Cmax) were calculated and expressed as mean ± standard error from five different experiments. ANOVA and Student’s *t*-test were selected as statistical analysis, *P* < 0.05 was considered representative of significant statistical differences.

### Cell Culture

Normal human epidermal melanocytes (NHEM) were purchased from Lonza (Walkersville, MD, United States) and were grown in Melanocyte growth medium 2 (Lonza). B16/F10 was purchased from IRCCS AOU San Martino – IST (Genova, Italy) and A375 was purchased from Sigma-Aldrich (Milan, Italy). Both were cultured in Dulbecco’s modified Eagle’s medium (DMEM) containing 10% FBS, 2 mmol/L L-glutamine, 100 μmol/L non-essential amino acids, penicillin (100 U/mL), streptomycin (100 μg/mL) and 1 mmol/L sodium pyruvate (all from Sigma-Aldrich, Milan, Italy). Cells were grown at 37°C in a humidified incubator under 5% CO_2_.

### MTT Assay

Cell proliferation was measured by 3-(4,3-(4,5-dimethylthiazol-2-yl)-2,5 diphenyltetrazolium bromide 5-dimethylthiazol-2-yl)-2, 5-diphenyltetrazolium bromide (MTT) assay. A375, B16F10 and NHEM cells were seeded on 96-well plates (2 × 10^3^ cells/well) and treated with NAP-HBTA (10-30-100 μM), HBTA or naproxen (100 μM) for 24-48-72 h. Then, medium was removed and replaced by 200 μL of 0.25 mg/mL MTT in 10% FBS-DMEM. After 3 h incubation, the reduced MTT dye was solubilized in 200 μL/well of DMSO. Absorbance was determined with a microplate spectrophotometer (Multiskan^TM^ GO Microplate Spectrophotometer) at 490 nm.

### Bromodeoxyuridine (BrdU) Incorporation Assay

Cell proliferation rate at 24-48-72 h after treatment with NAP-HBTA (10-30-100 μM) was measured by BrdU incorporation using the Cell Proliferation ELISA, BrdU (colorimetric) kit (Roche Diagnostics, Germany) according to the manufacturer’s instructions.

### Apoptosis Assay

Apoptosis was detected with annexin V-FITC assay (BD Pharmingen, San Diego, CA, United States) according to manufacturer’s instructions. A375 cells were seeded in 35 mm culture dishes and allowed to attach overnight. The cells were treated with NAP-HBTA (100 μM) for 24-48-72 h. To detect early and late apoptosis, both adherent and floating cells were harvested together, washed twice with PBS and resuspended in annexin V binding buffer at a concentration of 10^6^ cells/mL. Subsequently, 5 μL of FITC-conjugatedAnnexin V and 5 μL of PI were added to 100 μL of the cell suspension (10^5^ cells). The cells were incubated for 20 min at room temperature in the dark and subsequently analyzed using a two-laser equipped FACSCalibur apparatus and the CellQuest analysis software (Becton Dickinson, Mountain View, CA, United States).

### Western Blot Analysis

Whole-cell extracts were prepared from A375 cells treated with NAP-HBTA 100 μM or from melanoma tissue homogenate, as previously described (Panza et al., 2015). The protein concentration was measured by the Bradford method (Bio-Rad, Milan, Italy). Equal amounts of protein (40 μg/sample) were separated by SDS-PAGE and blotted onto nitrocellulose membranes (Trans-Blot Turbo Transfer Starter System, Biorad). The membranes were blocked for 2 h in 5% low-fat milk in PBS with 0.1% Tween 20 (PBST) at room temperature. Then the filters were incubated with the following primary antibodies: caspase 3 and PARP (Cell Signaling, United States; diluted 1:1000), MMP-2 and MMP-13 (Santa Cruz Biotechnology, Santa Cruz, CA, United States; diluted 1:1000), overnight at 4°C. The membranes were washed three times with PBST and then incubated with horseradish peroxidase-conjugated antibodies (Santa Cruz Biotechnology, Santa Cruz, CA, United States; diluted 1:2000) for 2 h at room temperature. The immune complexes were visualized by the ECL chemiluminescence method and acquired by the Image Quant 400 system (GE Healthcare).

### Wound Healing Assay

A375 or B16F10 cells were seeded in 12-well plates (2 × 10^5^ cells/well). Once the cells reached 90% confluency, a “wound” was made by manually scraping the middle of cell monolayers with a standard 200 μL pipette tip. After being washed three times with PBS, scratches including the flanking front lines of cells, were photographed (20-fold magnification). Subsequently, the cells were incubated in fresh complete medium with or without NAP-HBTA 10 or 30 μM. The width of the wound area was monitored with an inverted microscope at various time points and measured using Image J software (LASV3.8, Germany). Experiments were performed independently two times, evaluating 4–8 scratches in each experiment.

### Invasion Assay

The assay was performed using chambers with polycarbonate filters with 8-μm nominal pore size (Millipore, United States) coated on the upper side with Matrigel (Becton Dickinson Labware, United States). Briefly, the chambers were placed into a 24-well plate and A375 or B16F10 cells (2.5 × 10^5^/mL) were plated in the upper chamber with or without NAP-HBTA (10 or 30 μM in serum-free DMEM). After the incubation period (16 h), the filter was removed, and non-migrant cells on the upper side of the filter were detached with the use of a cotton swab. Filters were fixed with 4% formaldehyde for 15 min, and cells located in the lower filter were stained with 0.1% crystal violet for 20 min and then washed with PBS. The filters were examined microscopically and cellular invasion was determined by counting the number of stained cells on each filter in at least 4–5 randomly selected fields. Resultant data are presented as mean of invaded cells ± SEM/microscopic field of three independent experiments.

### Clonogenic Assay

A375 or B16F10 cells (1 × 10^3^ cells/well) were seeded in 6-well plates and treated with NAP-HBTA 10 or 30 μM for 48 h. After, fresh medium (drug-free) was changed every 2 days. Cells were cultured for 14 days to allow the colonies to form. Formed colonies were washed twice with 1x PBS, fixed by 4% paraformaldehyde, and stained with 0.5% crystal violet and colonies containing more than 50 cells (established by microscopy) were counted manually. Images of the colonies were obtained using a digital camera. The experiments were done in duplicate at least three times.

### ELISA

CXCL1/KC plasma concentrations were evaluated using ELISA kits according to the manufacturer’s instruction (DuoSet ELISA, R&D Systems, Minneapolis, MN, United States).

### Animals

Female C57BL/6 mice (18–20 g), 6–8 week-old, were purchased from Charles River Laboratories, Inc. (Germany). The experimental procedures were approved by the Italian Ministry in accordance with Italian (DL 26/2014) and European (Directive 2010/63/EU) regulations on the protection of animals used for experimental and other scientific purposes. Animal studies are reported in compliance with the ARRIVE guidelines (Kilkenny et al., 2010; McGrath and Lilley, 2015). All the animals were housed at the Animal Research Facility of the Department of Pharmacy of the University of Naples Federico II. They were subdivided in groups of five animals in clear transparent plastic cages with autoclaved dust free sawdust bedding. They were fed a pelleted and had unrestricted access to food and drinking water. The light/dark cycle in the room consisted of 12/12 h with artificial light. Mice were killed by CO_2_ inhalation.

### Induction of Subcutaneous B16 Lesions

The B16F10 syngeneic murine melanoma model has been widely used to study the mechanisms of melanoma development and progression and to evaluate the effect of any candidate drug (McKinney and Holmen, 2011). B16F10 murine melanoma cells (1 × 10^5^) in 100 μL saline were injected s.c. into the right flank of C57BL/6 mice (7 weeks-old). Animals were randomly divided into three groups (*n* = 8 each): one control group and two treatment groups. The control group was treated with vehicle (0.5% carboxymethyl cellulose/0.1% DMSO in double distilled water) whereas the other groups received NAP-HBTA (14.5 mg/kg) or naproxen (10 mg/kg) by oral gavage. Treatments were started immediately after the injection of the tumor cells and continued until day 14. All efforts were made to minimize suffering. Mice were observed daily and humanely euthanized by CO_2_ inhalation if a solitary subcutaneous tumor exceeded 1.5 cm in diameter or mice showed signs referable to metastatic cancer. Tumor sizes were measured using a digital caliper and tumor volumes were calculated using the following equation: tumor volume = π/6(D1xD2xD3) where D1 = length; D2 = width; D3 = height and expressed as cm^3^.

### Statistical Analysis

Data are expressed as mean ± SEM. Statistical analysis was performed with a number of *n* ≥ 3. Data were analyzed with GrapdPad Prism 6.0 software program (GraphPad Software, Inc., San Diego, CA, United States). One-way ANOVA was used for all of the statistical analyses among multiple groups. In another set of data the unpaired two tailed Student’s test was used for statistical analyses. A value of *P*-values < 0.05 was considered statistically significant.

## Results

### Chemistry

**Figure 1:**
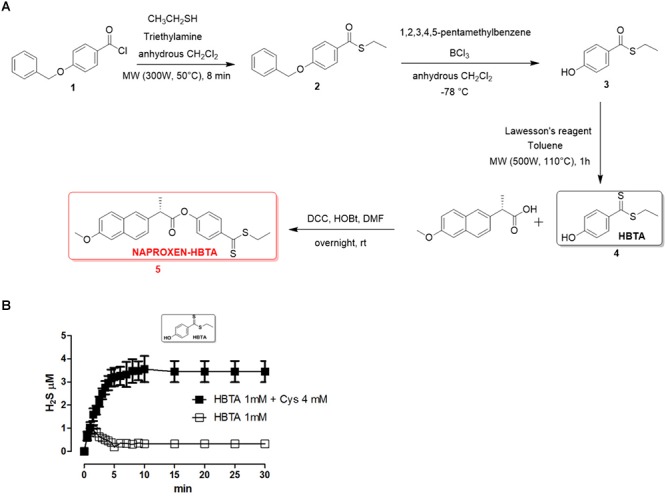
Synthetic procedure for the synthesis of naproxen-HBTA (**A**, **5**). H_2_S-releasing properties of HBTA evaluated by an amperometric approach. Curve describes the increase of H_2_S concentration, with respect to time, following the incubation of HBTA (1 mM) in the assay buffer with (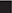) or without (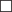) the nucleophile L-cysteine. H_2_S was recorded by amperometry; the vertical bars indicate the SEM **(B)**.

Naproxen-HBTA (**5,** Figure 1A) has been synthesized by esterification of commercially available naproxen (Sigma-Aldrich^®^) with ethyl 4-hydroxybenzodithioate (HBTA, **4,** Figure 1A), a compound identified by our research group as a new efficient H_2_S donor and described for this purpose for the first time in this paper. In particular, the synthetic procedure for the synthesis of the compound **5**, summarized in Figure 1, is as follows: thioesterification of 4-benzyloxybenzoylchloride (**1**) was achieved under microwave irradiation in 8 min treating the compound with a moderate excess of ethanethiol (1,5 equiv.) in presence of triethylamine in anhydrous dichloromethane giving in near quantitative yields the intermediate **2**. This compound was deprotected on the phenolic function following with modifications a previously published procedure (Okano et al., 2008) based on the reaction of the intermediate **2** with very large excess of BCl_3_ (8 equiv.) and pentamethylbenzene (10 equiv.) in anhydrous dichlorometane at -78°C. In this case the stochiometry of the reaction proved to be very important, because lower amounts of the reagents dramatically reduced the yield of the desired intermediate **3**. Conversion of intermediate **3** into a dithioester was efficiently performed by reaction with Lawesson’s reagent in toluene by microwave heating at 110°C to provide the novel H_2_S donor named HBTA (**4**, ethyl 4-hydroxybenzodithioate). Final coupling reaction with commercially available naproxen via DCC and HOBt in DMF afforded the desired compound **5.**

### *S*-Ethyl 4-(Benzyloxy)Benzothioate (2)

Commercially available 4-benzyloxybenzoylchloride (1) (Aldrich^®^, 1.5 g, 6.1 mmol) was dissolved in anhydrous dichloromethane (50 mL) in a two-neck flask and ethanethiol (0.568 g, 9.15 mmol) was added. The mixture was transferred into a sealed vessel and heated by microwave irradiation at 50°C (300 W power) for 8 min (30 s ramping step; 7 min and 30 s holding step). Solvent was evaporated under reduced pressure and the residue was purified by crystallization from n-hexane affording 1.64 g of intermediate **2**. Yield: 99% Anal. (C_16_H_16_O_2_S) C, H. m.p. 58–59°C ESI-MS: 273.2 [M + H]^+^; 295.1 [M+Na]^+^.

### *S*-Ethyl 4-Hydroxybenzothioate (3)

In a three neck flask and under nitrogen atmosphere, S-ethyl 4-(benzyloxy)benzothioate (**2,** 1.5 g, 5.5 mmol) was dissolved and stirred in anhydrous dichloromethane (20 mL). The mixture was cooled to -78°C and 1,2,3,4,5-pentamethylbenzene (8.15 g, 55 mmol), previously dissolved in 10 mL of anhydrous dichloromethane, was added dropwise. The reaction mixture was then further treated with a boron trichloride 1 M solution in dichloromethane (5.15 g, 44 mmol, 3.89 mL) that was added dropwise. The reaction mixture was stirred at -78°C for 45 min. Successively, 11 mL of a mixture of CHCl_3_/MeOH (10:1 v/v) was added and the stirring was prolonged for 5 min. The solvent was concentrated *in vacuo* and the residue was purified by column chromatography (CHCl_3_/hexane 1:1 (v/v)). The combined and evaporated product fractions used in the next step without any further purification. 0.90 g. Yield: 90% Anal. (C_9_H_10_O_2_S) C, H. m.p. 60–61°C ESI-MS: 183.1[M + H]^+1^H-NMR (DMSO-d_6_): δ d 10.42 (bs, 1H, OH), 7.76 (d, J = 5.0 Hz, 2H), 6.84 (d, J = 5.0 Hz, 2H), 2.96 (q, J = 4.5 Hz, 2H, CH_2_), 1.23 (t, J = 4.5 Hz, 3H, CH_3_) ^13^C-NMR (DMSO-d_6_): δ 189.68, 163.03, 129.68, 128.27, 115.97, 22.98, 15.38.

### *S*-Ethyl 4-Hydroxybenzodithioate (4, HBTA)

Intermediate **3** (0.45 g, 2.47 mmol) was dissolved in toluene (10 mL) and Lawesson’s reagent was added (662 mg, 1.48 mmol). The mixture was transferred into a sealed vessel and heated by microwave irradiation at 110°C (500 W power) for 1 h (5 min ramping step; 55 min holding step). The solvent was concentrated *in vacuo* and the residue purified by silica gel open column chromatography (DCM) to give 452 mg of the final compound as an orange oil. Yield: 92% Anal. (C_9_H_10_OS_2_) C, H. m.p. 48–49°C ESI-MS: 199.1 [M + H]^+^^1^H-NMR (DMSO-d_6_): δ 10.50 (bs, 1H, OH) 7.96 (d, J = 5.5 Hz, 2H), 6.81 (d, J = 5.5 Hz, 2H), 3.31 (q, J = 4.5 Hz, 2H, CH_2_), 1.29 (t, J = 4.5 Hz, 3H, CH_3_) ^13^C-NMR (DMSO-d_6_): δ 225.66, 163.26, 136.42, 129.45, 115.67, 30.84, 12.92.

The H_2_S-releasing properties of HBTA have been evaluated by an amperometric approach. The incubation of the selected compound in the assay buffer led to a fully negligible release of H_2_S; in contrast, in the presence of 4 mM L-cysteine, HBTA (1 mM) exhibited a slow and significant release of H_2_S (Figure 1B).

### *S*-4-((Ethylthio)Carbonothioyl)Phenyl 2-(6-Methoxynaphthalen-2-Yl)Propanoate (5, NAPROXEN-HBTA)

Intermediate **4** (0.195 g, 0.98 mmol), naproxen (Aldrich, 0.227 g, 0.98 mmol), *N-N*′- dicyclohexylcarbodiimide (0.223 g, 1.08 mmol) and 1-hydroxybenzotriazole (0.146 g, 1.08 mmol) were dissolved in dry DMF (10 mL) and stirred at room temperature for 4 h. The mixture was filtered off to remove DCU and the solvent was concentrated *in vacuo*. The residue was purified by silica gel open column chromatography (DCM) to give 233 mg of the final compound as an intese pink powder. Yield: 58% Anal. (C_23_H_22_O_3_S_2_) C, H. m.p. 103–105°C°C ESI-MS: 410.8 [M + H]^+^^1^H-NMR (DMSO-d_6_): δ 7.98 (d, J = 5.5 Hz, 2H,) 7.77–7.14 (m, 6H), 7.01 (d, J = 5.5 Hz, 2H), 4.10 (q, J = 4.5 Hz, 1H, CH), 3.93 (s, 3H, OCH_3_), 3.34 (q, J = 4.5 Hz, 2H, CH_2_), 1.70 (d, J = 4.5 Hz, 3H, CH_3_), 1.39 (t, J = 4.5 Hz, 3H, CH_3_). ^13^C-NMR (DMSO-d_6_): δ 227.17, 172.86, 157.80, 154.49, 142.38, 135.42, 133.94, 129.71, 128.94, 128.36, 127.76, 126.63, 126.36, 122.30, 119.35, 106.23, 55.66, 44.97, 31.60, 18.78, 12.55.

### Naproxen-HBTA Inhibits A375 and B16F10 Melanoma Cells Proliferation

**Figure 2:**
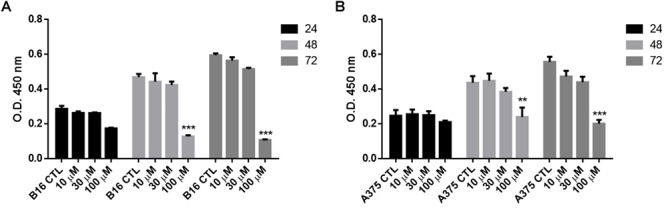
Effect of NAP-HBTA on **(A)** B16F10 and **(B)** A375 cell proliferation measured by the BrdU assay. (^∗∗^*P* < 0.01, ^∗∗∗^*P* < 0.001 vs. CTL).

The anti-proliferative effect of naproxen-HBTA was investigated on human melanoma cells A375 and murine melanoma cells B16F10. Cells were incubate with naproxen-HBTA (10-30-100 μM) or HBTA or naproxene alone (100 μM) for 24–48 and 72 h and cell growth was measured by the MTT assay. As shown in Table 1, a time- and concentration-dependent inhibition of cell growth was observed on both A375 and B16F10 cells. Non-tumorigenic cell line NHEM were used as negative control. In particular, cell proliferation 72 h following incubation with naproxen-HBTA (10-30-100 μM) was reduced by 14, 20, and 43%, respectively (*P* < 0.001) for A375 and by 11.5, 18, and 54%, respectively (*P* < 0.001) for B16F10 (Table 1). However, HBTA or naproxen alone did not affect A375 nor B16F10 cell proliferation at the maximum concentration tested (Table 1) suggesting that the synergism of naproxen with HBTA was the only responsible for the anti-proliferative effect on melanoma cells. In addition, the BrdU assay was performed to better confirm the anti-proliferative effect elicited by naproxen-HBTA. Results showed a greater inhibition of cell proliferation of both A375 and B16F10 cell lines (54 and 72% at 72 h respectively, *P* < 0.001) (Figure 2A,B).

**Table 1:** Effect of NAP-HBTA, HBTA and naproxen on A375, B16F10 and NHEM cell proliferation.

Cell line	CTRL	NAP-HBTA 10 μM	NAP-HBTA 30 μM	NAP-HBTA 100 μM	NAPROXEN 100 μM	HBTA 100 μM
**(A) 24**
NHEM	0.191 ± 0.003	0.191 ± 0.001	0.195 ± 0.005	0.189 ± 0.001	0.195 ± 0.002	0.195 ± 0.003
A375	0.252 ± 0.007	0.246 ± 0.001	0.240 ± 0.001	0.238 ± 0.001	0.300 ± 0.010	0.242 ± 0.010
B16/F10	0.383 ± 0.005	0.392 ± 0.002	0.430 ± 0.002	0.478 ± 0.006	0.399 ± 0.005	0.379 ± 0.002
**(B) 48**
NHEM	0.211 ± 0.001	0.209 ± 0.001	0.209 ± 0.002	0.212 ± 0.002	0.200 ± 0.002	0.212 ± 0.002
A375	0.480 ± 0.009	0.448 ± 0.020	0.440 ± 0.004	0.297 ± 0.020 ^∗∗^	0.496 ± 0.010	0.394 ± 0.030
B16/F10	0.877 ± 0.004	0.719 ± 0.004 ^∗∗^	0.713 ± 0.008 ^∗∗^	0.564 ± 0.006 ^∗∗∗^	0.883 ± 0.001	0.819 ± 0.001
**(C) 72**
NHEM	0.230 ± 0.006	0.233 ± 0.001	0.223 ± 0.002	0.219 ± 0.006	0.232 ± 0.002	0.224 ± 0.002
A375	0.634 ± 0.010	0.547 ± 0.020 ^∗∗^	0.509 ± 0.010 ^∗∗∗^	0.363 ± 0.008 ^∗∗∗^	0.671 ± 0.030	0.652 ± 0.030
B16/F10	1.016 ± 0.020	0.899 ± 0.005 ^∗∗^	0.832 ± 0.004 ^∗∗∗^	0.426 ± 0.007 ^∗∗∗^	0.986 ± 0.080	1.040 ± 0.030

*Growth inhibition was measured using the MTT assay and is expressed as OD values at 24-48-72 h. Only naproxen-HBTA (NAP-HBTA) inhibited the growth of all melanoma cells. NHEM were reported as negative control. Results were generated with three biological replicates each of them performed in technical quadruplicate (^∗∗^*P* < 0.01; ^∗∗∗^*P* < 0.001 vs. control CRTL)*.

### Naproxen-HBTA Induces Apoptosis of Human Melanoma Cells

**Figure 3:**
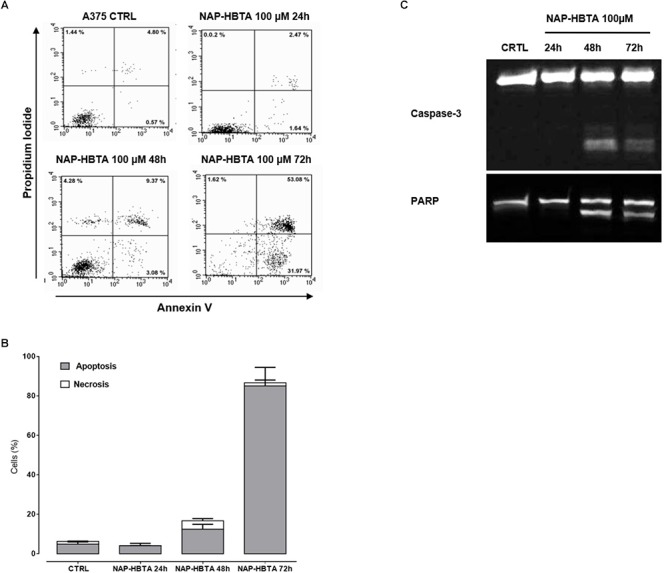
Naproxen-HBTA induces apoptosis of A375 human melanoma cells. Cells were treated with naproxen-HBTA (NAP-HBTA) (100 μM) at different time points and apoptosis was determined by annexin V/propidium iodide staining. Treatment of A375 cells for 24, 48, or 72 h with NAP-HBTA resulted in a time-dependent induction of apoptosis. Representative FACS plot and the relative quantitative analysis of apoptosis at various time points **(A,B)**. Western blot analysis of caspase 3 and PARP in whole-cell lysates. A375 cells were incubated with NAP-HBTA (100 μM) for 24, 48, or 72 h and a time-dependent cleavage of caspase 3 and of its substrate PARP was observed **(C)**. Experiments (*n* = 3) were performed in triplicate.

The inhibition of cell proliferation induced by naproxen-HTBA was consequent to induction of apoptosis as demonstrated by cytofluorimetric analysis. Naproxen-HBTA (100 μM) added to cells for 24–48–72 h provoked a significant time-dependent induction of apoptosis reaching the highest value, 85% of early and late apoptosis, at 72 h (Figure 3A,B). The pro-apoptotic effect of naproxen-HBTA was also confirmed by the cleavage of caspase 3, the main effector caspase, and of its substrate poly (adenosine diphosphate-ribose) polymerase (PARP) (Figure 3C).

### Naproxen-HBTA Inhibits Melanoma Cells Metastatic Features

**Figure 4:**
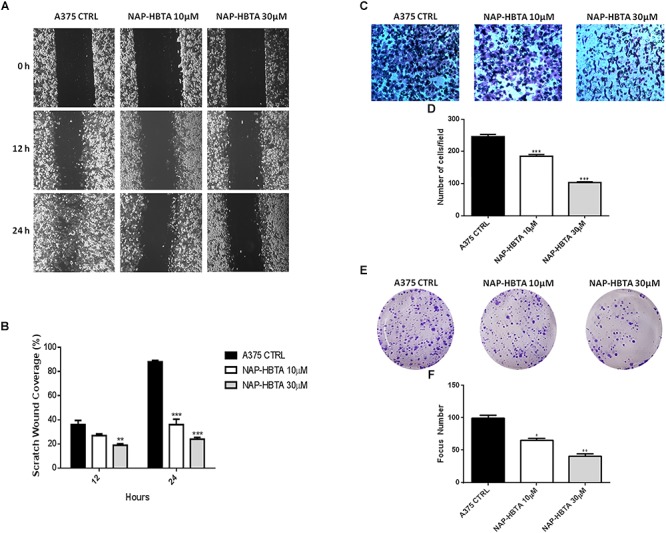
Naproxen-HBTA inhibits motility, invasiveness and cell colony formation of A375 human melanoma cells. A375 cells were treated with naproxen-HBTA (NAP-HBTA) (10 or 30 μM) for 12 or 24 h and the migration was determined by the rate of cells filling the scratched area. Representative photographs and average number of migratory A375 cells. **(A,B)** A375 cells were treated with naproxen-HBTA (NAP-HBTA) (10 or 30 μM) and cell invasivity was determined using boyden chambers coated with matrigel. Representative photographs and average number of invasive A375 cells. **(C,D)** A375 cells were treated with naproxen-HBTA (NAP-HBTA) (10 or 30 μM) and allowed for 14 days to form colonies. Representative photographs and average number of A375 colonies. **(E,F)** Treatment of A375 cells with NAP-HBTA resulted in a significant reduction of cell migration, invasion and colony formation. Data are shown as mean ± SEM of three independent experiments (^∗^*P* < 0.05, ^∗∗^*P* < 0.01, ^∗∗∗^*P* < 0.001 vs. CTRL).

Anti-tumor activity of naproxen-HBTA was further investigated by evaluating its ability to inhibit some of the malignant properties of melanoma cells. Migration and invasion of tumor cells are essential steps in the metastasis sequence (Obenauf and Massague, 2015). We used a classic wound healing assay to mimic to some extent migration of cells *in vivo*. Addition to A375 cells of naproxen-HBTA 10 or 30 μM (sub-IC_50_ concentration) at 0, 12, and 24 h inhibited cell migration at 12 h (*P* < 0.01) up to 24 h (*P* < 0.001) in a concentration-dependent manner (Figure 4A,B). To determine if naproxen-HBTA affected also the invasive potential of these melanoma cells an *in vitro* boyden chamber invasion assay was performed. Naproxen-HBTA was extremely effective in suppressing the invasive potential of A375 cells reducing significantly the numbers of invaded cells passing through the matrigel by 25% at 10 μM (185 ± 5.2 vs. control group: 246 ± 6.9, *P* < 0.05) and by 58% at 30 μM (103 ± 2.3 vs. control group: 246 ± 6.9, *P* < 0.001) (Figure 4C,D). Finally, to extend our understanding on the effects of naproxen-HBTA on melanocytic transformation we evaluated its ability to reduce the clonogenic potential of human melanoma cells, which correlates with the capacity of cells to produce progeny and tumor formation *in vivo* (Franken et al., 2006). After 48 h exposure to naproxen-HBTA (10–30 μM) the treated cells were cultured in drug-free medium for 14 days. As shown in Figure 4E,F, naproxen-HBTA significantly reduces the number of colonies about 30% (10 μM) (*P* < 0.05) and about 50% (30 μM) (*P* < 0.001) as well as focus diameter. All these data indicated that naproxen-HBTA significantly reduces all the proliferative, migratory and invasive properties of human melanoma cells. Similar results were also obtained with B16F10 murine melanoma cells (Supplementary Figure S1).

### Naproxen-HBTA Inhibits Melanoma Tumors Growth *in vivo*

**Figure 5:**
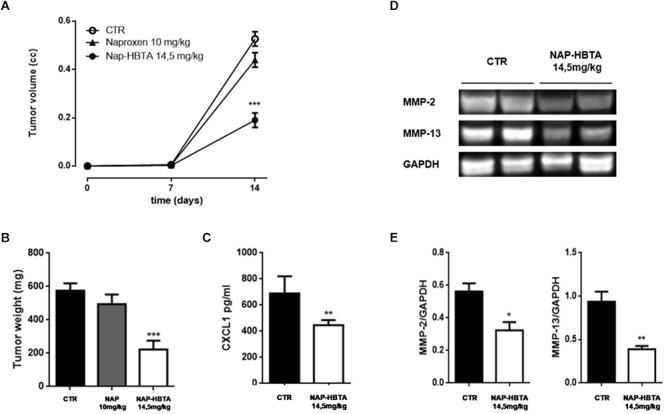
Naproxen-HBTA inhibits metastatic features in a syngeneic melanoma model *in vivo*. Naproxen-HBTA (NAP-HBTA) (14 mg/kg, 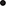), naproxen (10 mg/kg, 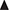), or vehicle (CTR, 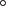) were administered orally every day for 14 days. Tumor volume was monitored on the indicated days. The average of tumor volume and tumor weight with standard error is showed respectively in **(A,B)**. In mice treated with NAP-HBTA tumor volume and tumor weight was significantly reduced (^∗∗∗^*P* < 0.001 vs. CTR, *n* = 8; day 14) **(A,B)**. CXCL1 plasma levels were significantly reduced in NAP-HBTA-treated mice (^∗∗^*P* < 0.01 vs. CTR, *n* = 8; day 14) **(C)**. Representative western blot analysis **(D)** and relative densitometry **(E)** of MMP-2 and MMP-13 proteins carried out on homogenate of subcutaneous melanoma excised from NAP-HBTA treated-mice or vehicle (CTR). In NAP-HBTA treated-mice the expression of both proteins was significantly reduced; GAPDH was detected as a loading control (^∗^*P* < 0.05; ^∗∗^*P* < 0.01 vs. CTR).

The interaction of cancer cells with the tumor microenvironment plays a major role in the function and regulation of cancer cells and is therefore a critical determinant of the response of cancer cells to therapeutic agents. Thus, to evaluate the clinical potential of naproxen-HBTA in melanoma treatment we used a well-known murine model of cutaneous melanoma. Tumor-bearing mice were treated twice daily with vehicle or naproxen-HBTA (14.5 mg/kg) or the parental drug naproxen (10 mg/kg). Naproxen-HBTA significantly reduced tumor volume by 64% (0.190 ± 0.03 cm^3^; *P* < 0.001) and tumor weight by 61% (222 ± 52.9 mg; *P* < 0.001) as compared to control mice (0.526 ± 0.03 cm^3^ and 575 ± 44 mg) (Figure 5A,B). As expected naproxen did not show any significant effect on tumor volume or weight (0.439 ± 0.03 cm^3^ and 494.5 ± 57.7 mg).

To further corroborate the reduction of metastatic features of melanoma tumor also *in vivo* we reported a significant reduction of the pro-metastatic chemokine CXCL1 in the plasma of naproxen-HBTA treated mice as compared with vehicle-treated mice (Figure 5C). Essential steps in the metastatic process are the angiogenesis process and remodeling of the extracellular matrix (ECM) by proteolytic enzymes such as matrix metalloproteinases (MMPs) which facilities the motility and the invasivity of tumor cells (Zhao et al., 2015). Melanoma cells express a number of various types of MMPs such as MMP-1, MMP-2, and MT1-MMP which have essential parts in melanoma vasculogenic mimicry formation (Seftor et al., 2001) and MMP-13 which promotes invasion and metastasis (Zhao et al., 2015). In tissue homogenate of subcutaneous melanoma excised from NAP-HBTA treated-mice we found a significant reduction of both MMP-2 and MMP-13 proteins compared to control mice (Figure 5D,E).

## Discussion

Melanoma cell spreading to distant organs is still a challenge in melanoma therapy and is associated with poor survival. Metastatic melanoma is highly chemoresistant and there is actually no effective treatment, indeed 5-year survival does not exceed 15% in patients with metastatic disease.(Singh and Salama, 2016) Over 50% of melanomas carrying activating V600E mutations in BRAF (BRAF^V600E^), an oncogene known to be critical for melanoma proliferation and survival.(Davies et al., 2002) In fact, despite BRAF represents an attractive target for melanoma drug development, responses to BRAF inhibitors are often brief, and resistance rapidly emerges (Solit and Rosen, 2011). Further studies on new therapeutic molecules, delivery systems and combination therapies for melanoma are therefore required to overcome the drug resistance problem. It is now clear the concept that an inflammatory microenvironment promotes tumor development and progression and it offers numerous targets for novel cancer prevention and treatment strategies (Mantovani et al., 2008). Indeed, aspirin [acetylsalicylic acid (ASA)] and/or other NSAIDs have been shown to reduce risk of gastric (Ye et al., 2013), colon (Chan et al., 2012), breast (Swede et al., 2005), and prostate cancer (Salinas et al., 2010) in humans. However, the clinical studies have provided contradictory results regarding NSAIDs use and melanoma risk (Hu et al., 2014). Eventually, statistically significant reduction of melanoma incidence was only evident with a regular ASA (100 mg/die) use for 5 or more years (Gamba et al., 2013). Despite the chemopreventive effect, chronic ingestion of NSAIDs is associated with some rate of toxicity like gastrointestinal bleeding and hemorrhagic stroke. Recently, a novel class of NSAIDs compounds has been developed by combining traditional NSAIDs with a chemical moiety that donates hydrogen sulfide. These hybrid molecules demonstrated an intrinsic gastrointestinal and cardiovascular safety, though having the same anti-inflammatory activity as the parent NSAID (Wallace, 2007). NSAIDs that release H_2_S have also shown to be more effective agents for melanoma treatment (De Cicco et al., 2016). The underlying mechanisms for the anticancer activities of H_2_S have been continuously exploring and new roles of H_2_S in cancer biology have been emerging. H_2_S donors proved to be useful as anticancer agents thanks to their multiple effects like cell signaling pathways inhibition, cell cycle regulation, microRNAs regulation, cancer metabolism, and pH regulation (Lee and Deng, 2015).

In this study, we designed and efficiently prepared an innovative hybrid H_2_S-releasing compound composed by naproxen and a completely novel H_2_S donor named HBTA. Intermediates and the final compound were obtained by following an excellent synthetic scheme that provided high yields and purity, opening to the opportunity of using HBTA as an efficient H_2_S donor as described for the first time in this paper. Moreover, the newly obtained anti-inflammatory hybrid donor was designed to couple with synergistic effect the H_2_S antitumor mechanisms to the naproxen-mediated COXs inhibition. Thus, to test that the new H_2_S moiety also increased the antitumoral activity of the NSAID-derivative we carried out both *in vitro* and *in vivo* experiments.

We found that naproxen-HBTA inhibited the proliferation of human melanoma cell line A375 and murine melanoma cell line B16F10. The anti-proliferative effect was subsequent to the induction of the apoptotic process as demonstrated by cytofluorimetric studies and by the cleavage of caspase 3, the main “effector” caspase in the apoptotic pathway. Conversely, HBTA and naproxen alone resulted uneffective on melanoma cell growth when used at equimolar concentrations.

Distant metastasis remarkably worsens the prognoses of malignant melanoma patients. Initiation of melanoma transformation and metastasis formation is associated with a “phenotype-switching” model of melanoma tumor cells. Indeed, melanoma tumor cells shift between highly proliferative phenotype within the primary tumor to more slowly proliferating but highly migratory and invasive phenotype in a distant site (Hoek et al., 2008; Roesch et al., 2010) due to their plasticity and to microenvironmental signals, such as hypoxia (Holmquist et al., 2006) and inflammation (de Visser et al., 2006). A375 are metastatic melanoma cells carrying the BRAF^V600E^ mutation conferring a high migratory phenotype and extensive invasion activity (Lu et al., 2016). Usually, chemotherapeutic agents targeting highly mitotic cells are not very efficient on the invasive cell population and this might explain drug resistance and tumor reoccurrence following treatment (Mitchison, 2012). Thus, the inhibitory effect shown by naproxen-HBTA on some features of metastasis such as motility, invasiveness and focus formation assumes clinical relevancy offering a new approach in the design of drugs for metastatic melanoma management. To strengthen the evidences obtained *in vitro*, we designed a translational *in vivo* pre-clinical approach. We used a syngeneic animal model that recapitulates human cutaneous melanoma progression, the spontaneous C57BL/6-derived B16F10 murine melanoma cells (McKinney and Holmen, 2011). Treatment of mice with naproxen-HBTA (14.5 mg/kg) significantly reduced tumor volume and weight. Naproxen, given to animals at the equivalent dose present in naproxen-HBTA, only slightly reduced tumor volume confirming that the inhibitory effect on tumor development showed by the molecule is the result of a synergism between the combination of H_2_S delivery and inhibition of COX activity. Cutaneous melanoma is a highly invasive and metastatic tumor. An interaction between primary tumor cells and metastatic cells might exist during the multistep cascade of distant metastases, via soluble factors. CXCL1 is an important chemokine whose levels result up-regulated in melanoma and it is essential for the establishment and the maintenance of the tumoral potential of melanoma (Dhawan and Richmond, 2002). Thus, we investigated if the reduction of tumor volume induced by naproxen-HTBA could be related to a down-regulation of CXCL1 production. We found that CXCL1 plasma levels of tumor-bearing mice treated with naproxen-HBTA were significantly lower as compared to control mice suggesting a potential effect of this new molecule on the production of chemokines involved in the metastasis spreading of malignant melanoma. Metastasis formation from cutaneous melanoma involve MMPs which play a crucial role due to their ability to degrade and remodel ECM and basement membrane (Hofmann et al., 2000). Indeed, MMP-13 has been associated with metastasis and poor survival in patients with primary melanoma (Zhao et al., 2015). Our data show that treatment with naproxen-HBTA in mice with cutaneous melanoma was able to reduce the expression of MMP-2 and MMP-13 in tumor tissues confirming the efficacy of naproxen-HBTA in inhibiting the metastatic potential of B16F10 melanoma cells and the metastasis initiation in mice. However, further studies will be required to better confirm the anti-metastatic potential of naproxen-HBTA *in vivo*.

## Conclusion

Our data demonstrate that the combination of COX-inhibition and H_2_S-delivery by naproxen-HBTA may offer a promising alternative to existing therapies for preventing melanoma development and progression to metastasis. In addition, since naproxen has a well-characterized pharmacokinetic profile and its H_2_S-derivative has also demonstrated to be safer regards organ-related toxicity, the new agent naproxen-HBTA could be quickly introduced into clinical trials as its cognates already do (Wallace et al., 2018).

## Author Contributions

GE, PD, FrF, and FeF designed, performed the experiments, analyzed the data, and wrote the manuscript. IS, AC, FG and EM performed the experiments. ViC and VaC have performed the H2S amperometric determination. BS and GC revised critically the manuscript. AI provided intellectual contributions, supervised all the experiments, revised critically the manuscript, and gave final approval to the publication.

## Conflict of Interest Statement

After the conclusion of the peer review, the handling editor noticed and declared a collaboration and shared affiliation with two of the authors, VaC and ViC. These authors provided an additional analysis requested by a reviewer during review. They were later added as co-authors and identified to the editor after the provisional acceptance of the manuscript. Under these exceptional circumstances, the relationship did not affect the objectivity of the review process, and this situation is being declared for transparency. The remaining authors declare that the research was conducted in the absence of any commercial or financial relationships that could be construed as a potential conflict of interest.

## Supplementary Material

Figure S1 Naproxen-HBTA inhibits motility, invasiveness, and cell colony formation of B16F10 murine melanoma cells. B16F10 cells were treated with naproxen-HBTA (NAP-HBTA) (10 or 30 μM) for 12 or 24 h and the migration was determined by the rate of cells filling the scratched area. Representative photographs and average number of migratory B16F10 cells. **(A,B)** B16F10 cells were treated with naproxen-HBTA (NAP-HBTA) (10 or 30 μM) and cell invasivity was determined using boyden chambers coated with matrigel. Representative photographs and average number of invasive B16F10 cells. **(C,D)** B16F10 cells were treated with naproxen-HBTA (NAP-HBTA) (10 or 30 μM) and allowed for 14 days to form colonies. Representative photographs and average number of B16F10 colonies. **(E,F)** Treatment of A375 cells with NAP-HBTA resulted in a significant reduction of cell migration, invasion and colony formation. Data are shown as mean ± SEM of three independent experiments (^∗^*P* < 0.05, ^∗∗∗^*P* < 0.001 vs. CTRL).
