# Erratum: Anti-metastatic Properties of Naproxen-HBTA in a Murine Model of Cutaneous Melanoma

**DOI:** 10.3389/fphar.2019.00213

**Published:** 2019-02-26

**Authors:** Frontiers Pharmacology Editorial Office

**Affiliations:** Pharmacology Editorial Office, Frontiers Media SA, Lausanne, Switzerland

**Keywords:** cyclooxygenases (COXs), hydrogen sulfide (H2S), non-steroidal anti-inflammatory drugs (NSAIDs), melanoma, target-therapy, apoptosis

The statement declaring the relationship between the handling editor and two of the authors has been clarified for transparency of the process. The updated statement should read, in full:

After the conclusion of the peer review, the handling editor noticed and declared a collaboration and shared affiliation with two of the authors, VaC and ViC. These authors provided an additional analysis requested by a reviewer during review. They were later added as co-authors and identified to the editor after the provisional acceptance of the manuscript. Under these exceptional circumstances, the relationship did not affect the objectivity of the review process, and this situation is being declared for transparency.

The publisher apologizes for this oversight. The original article has been updated.

