# Supplementary figures and images for: Anti-metastatic Properties of Naproxen-HBTA in a Murine Model of Cutaneous Melanoma
Source: Front Pharmacol. 2019 Feb 8;10:66. doi: 10.3389/fphar.2019.00066 (PMC6376415; doi:10.3389/fphar.2019.00066)

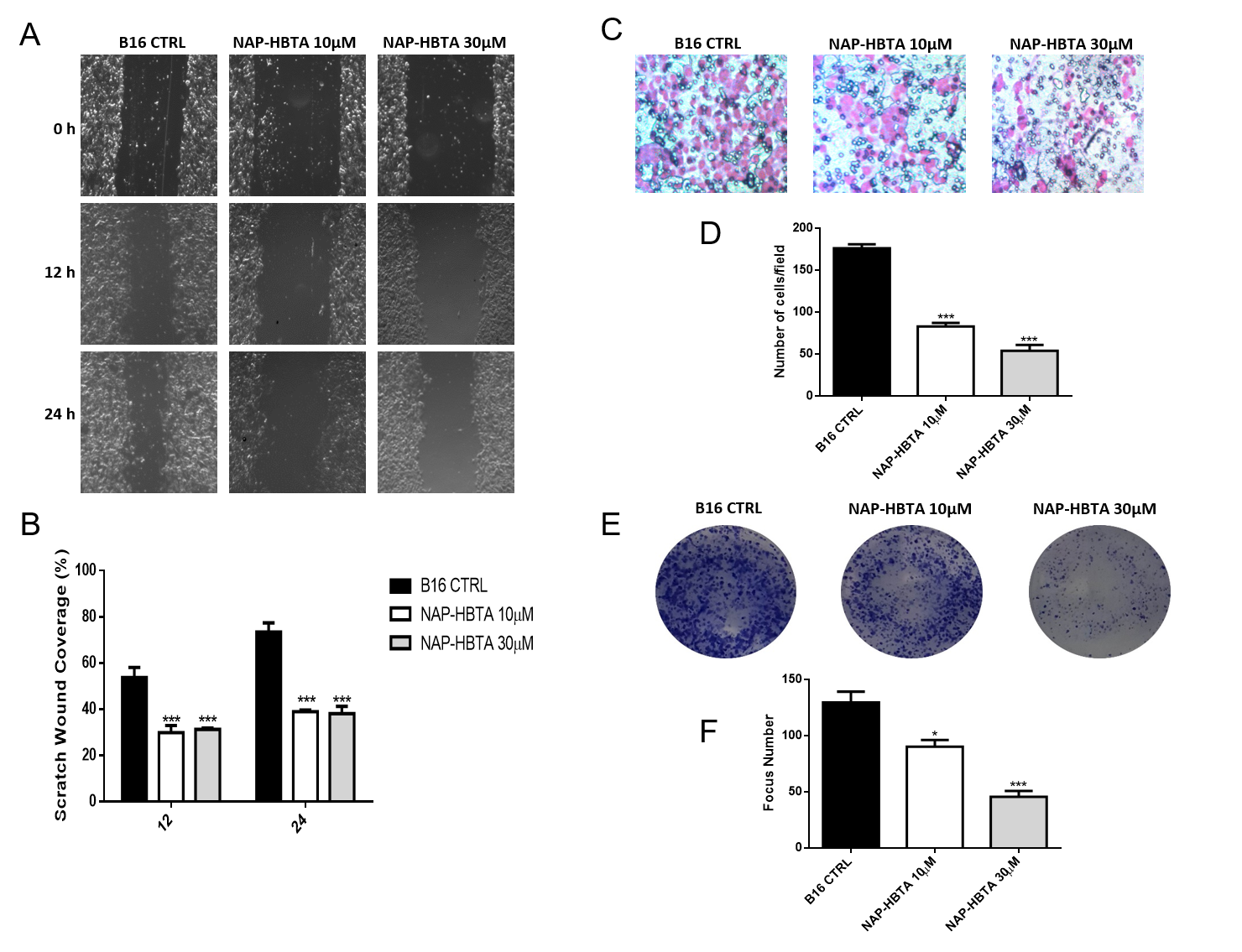

Supplement: Figure S1 — Naproxen-HBTA inhibits motility, invasiveness, and cell colony formation of B16F10 murine melanoma cells. B16F10 cells were treated with naproxen-HBTA (NAP-HBTA) (10 or 30 μM) for 12 or 24 h and the migration was determined by the rate of cells filling the scratched area. Representative photographs and average number of migratory B16F10 cells. (A,B) B16F10 cells were treated with naproxen-HBTA (NAP-HBTA) (10 or 30 μM) and cell invasivity was determined using boyden chambers coated with matrigel. Representative photographs and average number of invasive B16F10 cells. (C,D) B16F10 cells were treated with naproxen-HBTA (NAP-HBTA) (10 or 30 μM) and allowed for 14 days to form colonies. Representative photographs and average number of B16F10 colonies. (E,F) Treatment of A375 cells with NAP-HBTA resulted in a significant reduction of cell migration, invasion and colony formation. Data are shown as mean ± SEM of three independent experiments (∗P < 0.05, ∗∗∗P < 0.001 vs. CTRL). [file Image_1.TIF]
